# Electroacupuncture‐Induced Phrenic Nerve Stimulation for Poststroke Pneumonia: A Propensity Score Matching Analysis

**DOI:** 10.1155/bmri/6411927

**Published:** 2026-05-11

**Authors:** Bing-Feng Xing, Xiao-Qiang Huang, Hua Zhong, Qi-Fang Liang, Yang Chen, Rui Sun, Wei-Xiong Chen

**Affiliations:** ^1^ Department of Integrated Traditional Chinese and Western Medicine for Metabolic Diseases, The First Affiliated Hospital of Guangdong Pharmaceutical University, Guangzhou, China, gdpu.edu.cn; ^2^ Physical Inspection Section, The First Affiliated Hospital of Guangdong Pharmaceutical University, Guangzhou, China, gdpu.edu.cn

**Keywords:** diaphragmatic activity, electroacupuncture, phrenic nerve, pneumonia, stroke

## Abstract

**Background:**

Poststroke pneumonia (PSP) is a common complication in bedridden patients with speech dysfunction, often leading to poor outcomes. This retrospective cohort study explores the clinical efficacy of electroacupuncture (EA) by stimulating the phrenic nerve to improve diaphragmatic activity and treat PSP.

**Methods:**

In this study, 88 hospitalized poststroke patients with pneumonia were divided into two groups. The nonexposure group received standard treatment, including respiratory rehabilitation, whereas the exposure group received additional EA therapy targeting the phrenic nerve. Propensity score matching (PSM) was applied, resulting in 22 patients in the exposure group and 29 in the nonexposure group. Both groups were treated for 2 weeks, during which Clinical Pulmonary Infection Score (CPIS) scores, white blood cell (WBC) count, C‐reactive protein (CRP), interleukin‐6 (IL‐6), and procalcitonin (PCT) levels were measured. In the exposure group, diaphragmatic activity and thickness were also assessed before, during, and after EA.

**Results:**

The exposure group showed significantly greater improvements compared with the nonexposure group in reducing CPIS scores (mean difference: −1.087; 95% confidence intervals (CIs): −1.68 to −0.494; *p* < 0.05) and improving inflammatory markers (WBC, CRP, IL‐6, and PCT) (*p* < 0.05 for all comparisons). After PSM, CPIS scores demonstrated a larger absolute reduction in the exposure group compared with controls (−2.32 vs. −1.00), indicating a greater magnitude of clinical improvement. Within the exposure group, diaphragmatic activity improved significantly after 10 days of EA compared with baseline, with significant between‐group differences (*p* < 0.05 for all comparisons). Quantitative assessment further showed that diaphragmatic excursion increased by approximately 0.32 cm following intervention (95% CIs: 0.18–0.46), suggesting enhanced diaphragmatic functional mobility.

**Conclusion:**

EA may enhance diaphragmatic activity through phrenic nerve stimulation, improving ventilation, sputum clearance, and regulating neuroimmune responses, contributing to the effective management of PSP.

**Trial Registration:** International Traditional Medicine Clinical Trial Registry: ITMCTR2024000729

## 1. Introduction

Stroke, also known as cerebrovascular accident, has emerged as one of the leading causes of mortality worldwide due to its increasing incidence [1, 2]. In China, a comprehensive national survey conducted in 2019 revealed that stroke has overtaken all other diseases as the leading cause of death [3]. Stroke survivors often face a range of complex health conditions, which include motor dysfunction, dysphagia, restricted daily activities, urinary issues, hiccups, and prolonged immobility, all of which elevate the risk of complications during the recovery process [4–8]. Among these complications, poststroke pneumonia (PSP)—encompassing stroke‐associated pneumonia, aspiration pneumonia, and hospital‐acquired pneumonia—is one of the most prevalent and life‐threatening conditions in poststroke patients [9]. The rising incidence and mortality rates of PSP pose serious challenges to clinical management [10].

The overuse of antibiotics in managing PSP has led to the rise of antibiotic‐resistant bacteria, complicating the management of cardiopulmonary dysfunction and increasing the risk of secondary complications, such as venous thrombosis. These factors substantially impede patient recovery. Although current research on PSP remains limited, rehabilitation techniques such as airway clearance, respiratory training, and swallowing exercises have demonstrated potential in improving thoracic expansion, promoting mucus clearance, enhancing swallowing function to reduce aspiration, and increasing lung ventilation [11, 12]. However, many stroke patients experience cognitive impairments, diminished consciousness, speech dysfunction, or require tracheostomy care, making it challenging for them to actively engage in conventional rehabilitation programs. Consequently, there is a growing interest in passive and effective treatment modalities that require minimal patient involvement. This study seeks to address this gap by evaluating a passive treatment approach for PSP, with the goal of improving clinical outcomes.

Electroacupuncture (EA) has emerged as a potential therapeutic intervention that integrates traditional acupuncture with electrical stimulation to augment its therapeutic effects [13]. Studies have shown that EA can modulate the autonomic nervous system, improve neuromuscular function, and promote motor recovery, particularly in poststroke patients [9, 14]. One of the promising applications of EA in this context is its ability to stimulate the phrenic nerve, which plays a crucial role in controlling diaphragmatic movement, a key function for respiration [15, 16]. Diaphragmatic dysfunction is common in bedridden stroke patients, particularly those with weakened respiratory muscles, and it significantly contributes to the development of PSP [9, 17].

The potential mechanism of EA in PSP management lies in its ability to improve diaphragmatic function, thereby enhancing lung expansion and secretion clearance through phrenic nerve stimulation. This mechanism improves respiratory function by optimizing ventilation, promoting clearing mucus, and lowering pneumonia risk. EA has also been shown to regulate neuroimmune responses and reduce inflammation, both essential in managing infections such as pneumonia [18, 19]. These effects are especially relevant for stroke patients unable to participate in respiratory rehabilitation because of cognitive deficits, reduced consciousness, or tracheostomy. By passively improving diaphragmatic function, EA may serve as a valuable adjunct therapy for PSP [20].

This retrospective cohort study investigated the therapeutic potential of EA as an adjunct to standard respiratory treatments in patients with PSP. The primary objective was to assess whether EA‐mediated phrenic nerve stimulation could enhance diaphragmatic activity and improve clinical outcomes.

## 2. Materials and Methods

### 2.1. Study Design

This retrospective cohort study was a retrospective cohort analysis based on data collected from inpatients at the Department of Integrative Medicine, First Affiliated Hospital of Guangdong Pharmaceutical University, from January 1, 2023, to September 15, 2024. This study was approved by the Ethics Committee of the First Affiliated Hospital of Guangdong Pharmaceutical University (No: 2024‐IIT‐64), and all procedures were conducted in compliance with the Declaration of Helsinki and its amendments. Data were collected from the hospital′s electronic medical records, which included demographic characteristics, diagnostic codes, National Institutes of Health Stroke Scale (NIHSS) scores [21], Clinical Pulmonary Infection Score (CPIS) [22, 23], routine blood tests, and infection markers both at admission and after treatment. Propensity score matching (PSM) was performed to balance baseline differences between the treatment groups.

Propensity scores were estimated via logistic regression, incorporating baseline covariates known to influence PSP outcomes or treatment assignment: age, gender, stroke type (ischemic/hemorrhagic), NIHSS score at admission, stroke duration (categorized as 1–3 months or > 3 months), and key comorbidities (hypertension, diabetes, and smoking history). A 1:2 nearest‐neighbor matching protocol was prespecified to optimize statistical efficiency. However, the final matching ratio deviated from this target owing to the imposition of a strict caliper (0.01 standard deviations (SDs) of the logit propensity score) and the limited availability of eligible controls for certain treated patients. Specifically, not all exposed subjects could be successfully matched to two controls, resulting in a reduced final sample size while preserving covariate balance. Matching was performed without replacement within a caliper width of 0.01 SDs of the logit propensity score to ensure adequate similarity between groups.

### 2.2. Participants

The study participants above the ages of 18 who were diagnosed with pneumonia, in accordance with the “Chinese Expert Consensus on the Diagnosis and Treatment of Stroke‐Associated Pneumonia (2019 Update)” [24], were included in the study. All 88 eligible patients were initially assigned to one of two groups: the exposure group (*n* = 26), which received EA in combination with standard treatment and respiratory rehabilitation, and the nonexposure group (*n* = 62), which received only standard treatment and respiratory rehabilitation. Following PSM, 22 patients from the exposure group and 29 from the nonexposure group were matched and included in the final analysis. For patients who were not included in the PSM analysis, they were excluded using a direct deletion approach.

The treatment assignments were made based on clinical decisions in real‐world practice, with attending physicians collaborating with patients and their families to determine the most appropriate treatment plan. The decision to provide EA therapy in the exposure group was based on several real‐world factors: (1) the attending physician′s clinical judgment and familiarity with EA for respiratory rehabilitation; (2) informed preference of the patient or family after discussion of available treatment options; and (3) logistical feasibility of administering daily EA sessions. No formal randomization was used, consistent with the pragmatic, observational design of this study.

### 2.3. Eligibility Criteria

The eligibility criteria were defined to ensure uniformity across the study population.

#### 2.3.1. Inclusion Criteria

Diagnosis: Patients with a confirmed diagnosis of PSP according to the 2019 updated guidelines [24].

Stroke duration: Patients with a stroke history of more than 1 month, who were bedridden and exhibited speech or cognitive impairment. Patients with a stroke history of over 1 month were included to target those in the postacute to early chronic recovery phase. By this stage, the initial hyperacute management period has passed, yet patients remain at elevated risk for PSP due to persistent neurological deficits—such as being bedridden or having impaired speech and cognition. This selection criterion helped standardize the study population with respect to stroke recovery stage and PSP risk.

Age: Patients aged 18 years or older, regardless of gender.

Stroke severity: NIHSS score of 3 or higher.

Treatment duration: Patients who received respiratory rehabilitation and EA for at least two consecutive weeks.

#### 2.3.2. Exclusion Criteria

Progressive stroke: Patients with ongoing stroke progression or deterioration.

Other infections: Patients with special infections such as tuberculosis, H1N1, COVID‐19, avian flu, or other atypical pneumonias.

Pulmonary complications: Patients with bronchiectasis, chronic obstructive pulmonary disease, bronchial asthma, malignant lung diseases, or those requiring mechanical ventilation.

Mental health conditions: Patients diagnosed with psychiatric or psychological disorders.

Critical conditions: Patients with acute heart failure, shock, or other life‐threatening conditions.

#### 2.3.3. Data Exclusion Criteria

Transfer or discharge: Patients who were transferred to other hospitals or departments during treatment, resulting in missing posttreatment evaluation data.

Condition changes: Patients whose conditions changed during the study, making them ineligible based on either inclusion or exclusion criteria.

### 2.4. Interventions

All patients received treatment for more than 2 weeks as prescribed. The exposure group received respiratory rehabilitation and EA‐based phrenic nerve stimulation in addition to standard medical care, once daily for two consecutive weeks. EA treatment was initiated on the first day of the intervention period, with each treatment session lasting 30 min. Treatment was provided daily for 14 consecutive days. EA was administered to patients unable to participate in conventional rehabilitation due to cognitive deficits, reduced consciousness, or the need for tracheostomy care. This approach is aimed at providing a passive, noninvasive treatment option to improve clinical outcomes. Standard treatment included secondary stroke prevention, pneumonia treatment, nutritional support, and symptomatic care, whereas respiratory rehabilitation included airway clearance techniques, respiratory training, and swallowing exercises. The exposure group was treated with sterile acupuncture needles (0.25 × 25 mm and 0.25 × 40 mm; Medical Device Registration No.: SXZZ. 20162200588) from Huatuo, and an EA stimulator (SDZ‐V; Medical Device Registration No.: SXZZ. 20172200675). Acupuncture points included bilateral Tianding (LI 17, on the lateral neck, approximately 1 cun inferior to the posterior border of the mastoid process) and Wuyi (ST 5, on the face, directly below the pupil in forward gaze, at the lower border of the zygomatic bone, anterior to the masseter muscle where the facial artery pulsates). A discontinuous waveform was applied, with frequency set between 1 and 5 Hz, starting at 1 Hz, and intensity increased to a tolerable level.

**Figure 1:**
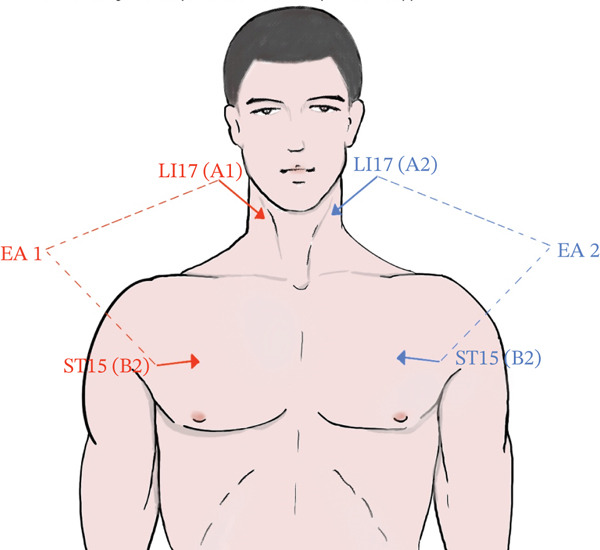
Schematic diagram of acupoints used in the electroacupuncture therapy.

EA treatment was performed with the patient lying flat on a treatment bed. After standard skin disinfection, needles were inserted perpendicularly at LI 17 (0.8–1.0 cun) and transversely at ST 5 (0.5–0.8 cun), with gentle manipulation to induce the deqi sensation (a feeling of aching, numbness, or distension, sometimes radiating sensations along the meridian). EA was applied to the same side at LI 17 and ST 5 (Figure 1). All treatments were administered by experienced acupuncturists.

### 2.5. Outcome Measures

#### 2.5.1. Efficacy Measures

1. Clinical efficacy: The CPIS score, which evaluates the severity of pneumonia using clinical and radiographic criteria, was used. The score includes assessments of body temperature, white blood cell (WBC) count, tracheal secretions, oxygenation, pulmonary infiltrates on X‐ray, and microbiological findings, with scores ranging from 0 to 2 for each parameter. Routine blood tests were performed using an automated blood analyzer (Mindray BC6800); blood gas analysis was conducted using a multiparameter blood gas analyzer (Roche cobas b221), and sputum cultures were incubated on blood agar, MacConkey agar, Sabouraud dextrose agar, and chocolate agar plates in a CO_2_ incubator. Chest X‐rays were obtained using a Philips digital X‐ray machine (Digital Diagnsot DR 1000 mA). All data were collected before and after 2 weeks of treatment.
2. Stroke severity: Baseline NIHSS scores were recorded for all participants prior to the intervention and used for group comparisons.
3. Inflammatory markers: Serum levels of C‐reactive protein (CRP), interleukin‐6 (IL‐6), and procalcitonin (PCT) were measured. IL‐6 and PCT were analyzed using an automated analyzer (Roche cobas e 601), with Roche IL‐6 and PCT assay kits. CRP was measured using an automated analyzer (Beckman AU5800) with a CRP assay kit (Sekisui Medical). Samples were collected before and after 2 weeks of treatment.
4. Diaphragmatic activity: Diaphragmatic activity was measured with the patient in a supine position using a Konica Minolta ultrasound machine (Fujifilm Investment Co. Ltd.) in 2D mode with an M‐mode convex array probe (2–5 MHz) to assess diaphragm movement and a linear probe (6–13 MHz) to measure diaphragm thickness. The probe was first placed perpendicular to the chest wall along the midaxillary line between the 8th and 11th intercostal spaces to measure diaphragm thickness. It was then repositioned along the anterior axillary line or the costal margin–midclavicular intersection, oriented inferolaterally, to measure diaphragm excursion. Three consecutive measurements were taken, and the average value was recorded. Data were collected before, during, and after 2 weeks of EA.

#### 2.5.2. Safety Measures

Adverse reactions to EA, such as needle‐related injuries, subcutaneous hematomas, abnormal respiratory rates, and heart rate fluctuations, were monitored and recorded throughout the study.

### 2.6. Quality Control and Bias Management

Potential selection bias inherent to this retrospective real‐world study was addressed through multiple strategies. First, strict inclusion and exclusion criteria were applied to ensure clinical comparability. Second, baseline demographic and clinical variables were extracted objectively from electronic medical records. Third, PSM was employed to balance key confounders between groups. To further strengthen the assessment of covariate balance following matching, standardized mean difference (SMD) was calculated for all variables included in the propensity score model. An SMD of < 0.1 was predefined as the threshold for acceptable balance, in line with established methodological recommendations. The postmatching SMD values confirmed that baseline covariates were well balanced between groups, thereby providing additional quantitative support for the robustness and adequacy of the matching procedure. Finally, postmatching comparisons confirmed adequate baseline balance, thereby minimizing selection bias.

Nevertheless, given the retrospective observational nature of the study, treatment allocation was influenced by real‐world clinical decision‐making, including physician judgment, patient or family preference, and practical treatment feasibility. Therefore, despite the application of PSM, residual confounding and indication bias cannot be completely excluded. Accordingly, the findings of this study should be interpreted as reflecting associative relationships rather than definitive causal effects.

### 2.7. Statistical Methods

Statistical analysis was conducted on all patients who met the inclusion criteria. PSM analysis was performed at a 1:2 ratio using a caliper value of 0.01. Continuous variables were reported as mean ± SD and analyzed using independent sample *t*‐tests. Categorical variables were reported as frequencies (*n*, %) and analyzed using chi‐square tests. For repeated measures data, including NIHSS scores and inflammatory markers, a two‐way repeated‐measures analysis of variance (ANOVA) was applied to evaluate temporal changes within and between groups. Specifically, the ANOVA model incorporated group (exposure vs. nonexposure) and time as fixed effects, with particular emphasis on the group × time interaction to determine whether the trajectory of change differed between groups over time. Where significant interaction effects were identified, appropriate post hoc analyses were conducted to further characterize between‐group differences at specific time points. In addition to hypothesis testing based on *p* values, we calculated absolute differences with their corresponding 95% confidence intervals (CIs). This approach provides a more nuanced quantification of treatment effects and enhances interpretability for clinical application. These estimates complement traditional significance testing by offering insight into the magnitude and precision of observed effects. All statistical analyses were conducted using SPSS Statistics 26.0.

## 3. Results

### 3.1. Patient Selection

**Figure 2:**
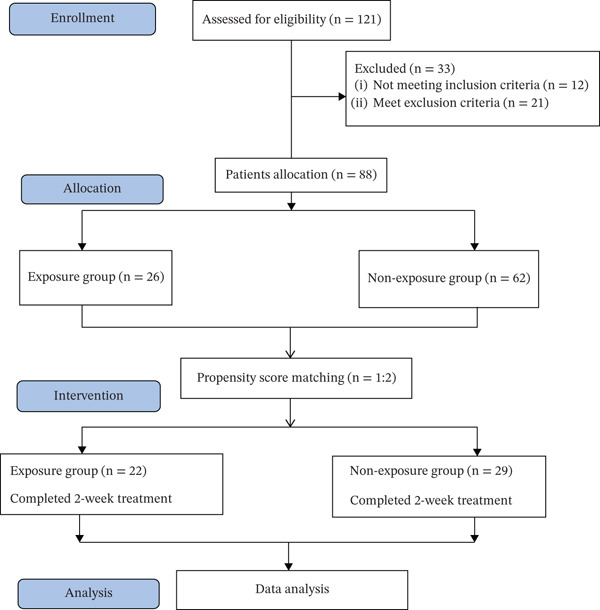
Flowchart of patient selection.

A total of 121 patients were initially assessed at the First Affiliated Hospital of Guangdong Pharmaceutical University in China. However, 33 patients were excluded from the study for the following reasons: 12 patients did not meet the inclusion criteria, and 21 patients were excluded based on the exclusion criteria. Thus, 88 eligible patients were included in the analysis and divided into two groups: the exposure group (*n* = 26) and the nonexposure group (*n* = 62). To minimize potential biases, PSM was applied with a ratio of 1:2. After matching, 22 patients in the exposure group and 29 patients in the nonexposure group completed the 2‐week treatment protocol and were included in the final analysis (Figure 2).

### 3.2. Baseline Characteristics of Patients

Baseline characteristics were compared before and after PSM. Before matching, significant differences were observed between the groups in stroke duration and NIHSS scores. After matching, these differences were no longer statistically significant, suggesting improved comparability between groups. In addition to conventional statistical comparisons, SMD metrics were calculated for all matched covariates to provide a sample size–independent assessment of balance. All postmatching SMD values were below the predefined threshold of 0.1, indicating adequate covariate balance between the exposure and nonexposure groups and supporting the effectiveness of the matching procedure.

Post‐PSM, the male‐to‐female ratio remained imbalanced, with a higher proportion of male patients in both groups. This imbalance reflects both the higher prevalence of male patients during the study period and the slightly higher incidence of stroke in men in the general population. In both groups, the proportion of male patients was higher than that of female patients, with a postmatching male‐to‐female ratio of approximately 1.4:1. The incidence of ischemic stroke was also higher than that of hemorrhagic stroke, with a postmatching ratio of approximately 1.2:1. Although residual sex imbalance persisted after matching, the overall baseline clinical characteristics between groups remained broadly comparable. Given the relatively small sample size following PSM, meaningful subgroup or interaction analyses based on sex were not statistically feasible. Therefore, the potential influence of sex‐related biological differences on treatment response cannot be fully excluded and should be interpreted with caution. Future studies with larger, adequately powered cohorts are warranted to further investigate possible sex‐specific differences in response to EA intervention.

Among the recorded comorbidities, hypertension was the most prevalent condition, followed by diabetes, hyperlipidemia, and hyperhomocysteinemia. More than 45% of patients were smokers and over 20% reported alcohol consumption (Tables 1 and 2).

**Table 1:** Baseline characteristics of patients.

	Before PSM					After PSM				
Items	Exposure group (*n* = 26)	95% CI	Nonexposed group (*n* = 62)	95% CIs	SMD	Exposure group (*n* = 22)	95% CIs	Nonexposed group (*n* = 29)	95% CIs	SMD
Age	69.65 ± 11.97	64.82–74.48	76.35 ± 10.94	73.63–79.07	0.584	69.45 ± 11.45	64.37–74.53	75.55 ± 10.76	71.46–79.64	0.549
Course of disease	5.35 ± 5.85	2.99–7.71	6.45 ± 4.16^∗∗^	5.41–7.49	0.217	5.01 ± 5.28	2.67–7.35	5.10 ± 3.43	3.80–6.40	0.020
NIHSS	11.11 ± 6.68	8.41–13.81	10.77 ± 5.28^∗^	9.46–12.08	0.056	10.45 ± 4.76	8.34–12.56	10.55 ± 5.02	8.64–12.46	0.020
CPIS	6.42 ± 1.36	5.87–6.97	6.35 ± 1.41	6.00–6.70	0.051	6.22 ± 1.34	5.63–6.81	6.17 ± 1.58	5.57–6.77	0.034
Gender										
Male	17 (65.38%)	47.10%–83.67%	37 (59.67%)	47.47%–71.89%	0.118	13 (59.09%)	38.55%–79.64%	17 (58.62%)	40.70%–76.55%	0.010
Female	9 (34.61%)	16.33%–52.90%	25 (40.32%)	28.11–52.53%	0.118	9 (40.09%)	20.36%–61.45%	12 (41.37%)	23.45%–59.30%	0.010
Type of stroke										
IS	14 (53.84%)	34.68%–73.01%	39 (62.9%)	50.88%–74.93%	0.185	12 (54.54%)	33.74%–75.35%	16 (55.17%)	37.07%–73.27%	0.013
HS	12 (46.15%)	26.99%–65.32%	23 (37.09%)	25.07%–49.12%		10 (45.45%)	24.65%–66.26%	13 (44.82%)	26.73%–62.93%	
Diabetes	12 (46.15%)	26.99%–65.32%	26 (41.93%)	29.65%–54.22%	0.085	9 (40.9%)	20.36%–61.45%	11 (37.93%)	20.27%–55.59%	0.061
Hypertension	21 (80.76%)	65.62%–95.92%	45 (72.58%)	61.48%–83.68%	0.194	19 (86.36%)	72.02%–100%	23 (79.31%)	64.57%–94.05%	0.188
Hyperlipidemia	7 (26.92%)	9.87%–43.97%	18 (29.03%)	17.73%–40.33%	0.047	6 (27.27%)	8.66%–45.88%	9 (31.03%)	14.20%–47.87%	0.083
Homocysteinemia	5 (19.23%)	4.08%–34.38%	11 (17.74%)	8.23%–27.25%	0.038	4 (18.18%)	2.06%–34.30%	7 (24.13%)	8.56%–39.71%	0.146
Smoke	12 (46.15%)	26.99%–65.32%	26 (41.93%)	29.65%–54.22%	0.085	10 (45.45%)	24.65%–66.26%	14 (48.27%)	30.09%–66.46%	0.057
Alcohol abuse	6 (23.07%)	6.88%–39.27%	17 (27.42%)	16.32%–38.52%	0.100	5 (22.72%)	5.22%–40.24%	8 (27.58%)	11.32%–43.85%	0.112

Abbreviations: CIs, confidence intervals; CPIS, Clinical Pulmonary Infection Score; HS, hemorrhagic stroke; IS, ischemic stroke; NIHSS, National Institutes of Health Stroke Scale; PSM, propensity score matching; SMDs, standardized mean differences.

^∗^ *p* < 0.05.

^∗∗^ *p* < 0.01.

**Table 2:** Baseline characteristics of patients.

	Before PSM				After PSM			
Items	Exposure group (*n* = 26)	95% CI	Nonexposed group (*n* = 62)	95% CI	Exposure group (*n* = 22)	95% CI	Nonexposed group (*n* = 29)	95% CI
Age	69.65 ± 11.97	64.82–74.48	76.35 ± 10.94	73.63–79.07	69.45 ± 11.45	64.37–74.53	75.55 ± 10.76	71.46–79.64
Course of disease	5.35 ± 5.85	2.99–7.71	6.45 ± 4.16^∗∗^	5.41–7.49	5.01 ± 5.28	2.67–7.35	5.10 ± 3.43	3.80–6.40
NIHSS	11.11 ± 6.68	8.41–13.81	10.77 ± 5.28^∗^	9.46–12.08	10.45 ± 4.76	8.34–12.56	10.55 ± 5.02	8.64–12.46
CPIS	6.42 ± 1.36	5.87–6.97	6.35 ± 1.41	6.00–6.70	6.22 ± 1.34	5.63–6.81	6.17 ± 1.58	5.57–6.77
Gender								
Male	17 (65.38%)	47.10%–83.67%	37 (59.67%)	47.47%–71.89%	13 (59.09%)	38.55%–79.64%	17 (58.62%)	40.70%–76.55%
Female	9 (34.61%)	16.33%–52.90%	25 (40.32%)	28.11–52.53%	9 (40.09%)	20.36%–61.45%	12 (41.37%)	23.45%–59.30%
Type of stroke								
IS	14 (53.84%)	34.68%–73.01%	39 (62.9%)	50.88%–74.93%	12 (54.54%)	33.74%–75.35%	16 (55.17%)	37.07%–73.27%
HS	12 (46.15%)	26.99%–65.32%	23 (37.09%)	25.07%–49.12%	10 (45.45%)	24.65%–66.26%	13 (44.82%)	26.73%–62.93%
Diabetes	12 (46.15%)	26.99%–65.32%	26 (41.93%)	29.65%–54.22%	9 (40.9%)	20.36%–61.45%	11 (37.93%)	20.27%–55.59%
Hypertension	21 (80.76%)	65.62%–95.92%	45 (72.58%)	61.48%–83.68%	19 (86.36%)	72.02%–100%	23 (79.31%)	64.57%–94.05%
Hyperlipidemia	7 (26.92%)	9.87%–43.97%	18 (29.03%)	17.73%–40.33%	6 (27.27%)	8.66%–45.88%	9 (31.03%)	14.20%–47.87%
Homocysteinemia	5 (19.23%)	4.08%–34.38%	11 (17.74%)	8.23%–27.25%	4 (18.18%)	2.06%–34.30%	7 (24.13%)	8.56%–39.71%
Smoke	12 (46.15%)	26.99%–65.32%	26 (41.93%)	29.65%–54.22%	10 (45.45%)	24.65%–66.26%	14 (48.27%)	30.09%–66.46%
Alcohol abuse	6 (23.07%)	6.88%–39.27%	17 (27.42%)	16.32%–38.52%	5 (22.72%)	5.22%–40.24%	8 (27.58%)	11.32%–43.85%

Abbreviations: CPIS, Clinical Pulmonary Infection Score; HS, hemorrhagic stroke; IS, ischemic stroke; NIHSS, National Institutes of Health Stroke Scale; PSM, propensity score matching.

^∗^ *p* < 0.05.

^∗∗^ *p* < 0.01.

### 3.3. CPIS Efficacy Evaluation

CPIS scores were assessed both within groups (pre–post comparisons) and between groups (intergroup differences) to provide a comprehensive evaluation of treatment effects. No significant differences were observed between groups at baseline (Table 3).

**Table 3:** Indicators before and after intervention in both groups.

	Exposure group (*n* = 22)				Nonexposed group (*n* = 29)			
Indicators	PRE	POST	Absolute difference	95% CIs	PRE	POST	Absolute difference	95% CIs
CPIS	6.22 ± 1.34	3.9 ± 0.81^∗^	−2.32	[−2.81, −1.83]	6.17 ± 1.58	5.17 ± 1.44	−1.00	[−1.55, −0.45]
WBC	12.65 ± 3.47	6.25 ± 2.00^∗^	−6.40	[−7.66, −5.14]	12.07 ± 3.18	8.55 ± 3.13	−3.52	[−4.67, −2.37]
CRP	33.30 ± 20.51	11.15 ± 12.85^∗^	−22.15	[−29.65, −14.65]	37.81 ± 28.56	30.79 ± 33.43	−7.02	[−18.40, 4.36]
IL‐6	29.93 ± 11.73	11.01 ± 7.12^∗^	−18.92	[−23.20, −14.64]	58.41 ± 12.84	19.14 ± 15.13^∗^	−39.27	[−44.41, −34.13]
PCT	0.507 ± 0.426	0.091 ± 0.107^∗∗^	−0.42	[−0.58, −0.26]	0.640 ± 1.418	0.261 ± 0.425	−0.38	[−0.84, 0.08]

*Note:* Absolute difference = POST − PRE. 95% CI calculated using paired *t*‐test method.

Abbreviations: CIs, confidence intervals; CPIS, Clinical Pulmonary Infection Score; CRP, C‐reactive protein (mg/L); IL‐6, interleukin‐6 (pg/mL); PCT, procalcitonin (ng/mL); POST, postintervention; PRE, preintervention; WBC, white blood cell count (×10^9^/L).

^∗^ *p* < 0.05.

^∗∗^ *p* < 0.01.

Within‐group analysis demonstrated that the exposure group experienced a statistically significant reduction in CPIS scores after treatment (absolute difference: −2.32; 95% CIs: −2.81 to −1.83; *p* < 0.05), indicating substantial improvement in pneumonia severity. In contrast, the nonexposure group showed a smaller and nonsignificant reduction (absolute difference: −1.00; 95% CIs: −1.55 to −0.45; *p* = 0.679).

Between‐group comparison further revealed that the magnitude of CPIS reduction was significantly greater in the exposure group than in the nonexposure group (mean difference: −1.087; 95% CIs: −1.68 to −0.494; *p* = 0.038), indicating a superior treatment effect associated with EA intervention.

**Figure 3:**
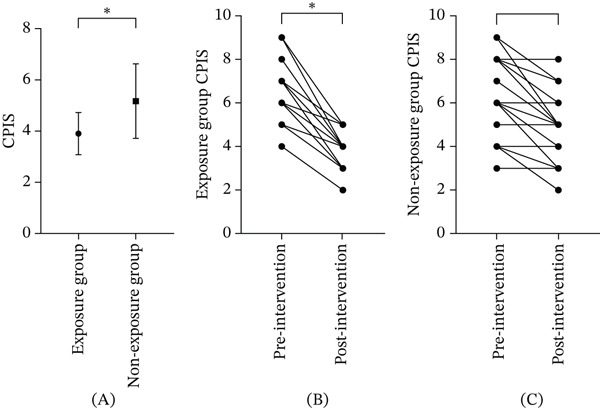
Changes in CPIS scores within and between the two groups.

Consistent with the repeated‐measures analytical framework, a significant group × time interaction effect was observed, suggesting that the trajectory of CPIS improvement differed significantly between groups. These findings are illustrated in Figure 3A–C.

### 3.4. WBC, CRP, IL‐6, and PCT

**Figure 4:**
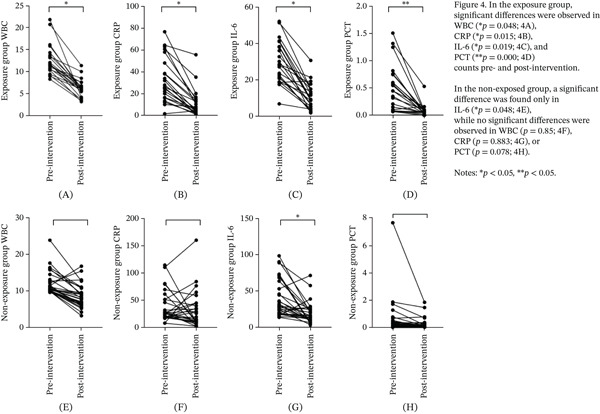
Changes in WBC, CRP, IL‐6, and PCT within groups.

**Figure 5:**
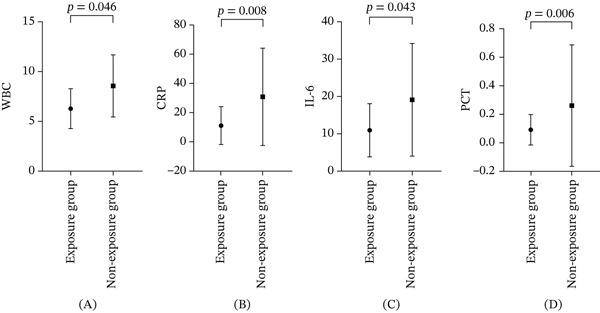
Changes in WBC, CRP, IL‐6, and PCT between the two groups.

The levels of WBC, CRP, IL‐6, and PCT were compared within and between the two groups before and after the intervention (Table 3). At baseline, no significant differences were detected in any marker between the groups. After the intervention, the exposure group showed significant reductions in all four biomarkers compared with preintervention levels: WBC (*p* = 0.048), CRP (*p* = 0.015), IL‐6 (*p* = 0.019), and PCT (*p* < 0.001) (Figure 4A–D). In contrast, the nonexposure group showed a significant reduction only in IL‐6 (*p* = 0.048), whereas no significant changes were observed in WBC (*p* = 0.85), CRP (*p* = 0.883), or PCT (*p* = 0.078) (Figure 4E–H). Effect size estimation further demonstrated clinically meaningful reductions in inflammatory biomarkers within the exposure group, including WBC (absolute difference: −6.40 × 10^9^/L; 95% CIs: −7.66 to −5.14), CRP (absolute difference: −22.15 mg/L; 95% CIs: −29.65 to −14.65), IL‐6 (absolute difference: −18.92 pg/mL; 95% CIs: −23.20 to −14.64), and PCT (absolute difference: −0.42 ng/mL; 95% CIs: −0.58 to −0.26). In comparison, reductions observed in the nonexposure group were generally smaller and accompanied by wider CIs, suggesting lower estimate precision and reduced treatment magnitude (Table 3). Postintervention, between‐group comparisons revealed significantly lower levels in the exposure group than in the nonexposure group for all markers: WBC (*p* = 0.046), CRP (*p* = 0.008), IL‐6 (*p* = 0.043), and PCT (*p* = 0.006) (Figure 5A–D). Importantly, within the repeated‐measures analytical framework, significant group × time interaction effects were identified for these inflammatory markers, indicating that the temporal trajectories of biomarker reduction differed significantly between groups. This finding further supports a treatment‐specific effect of EA beyond general time‐dependent recovery.

### 3.5. Diaphragmatic Activity Monitoring

**Figure 6:**
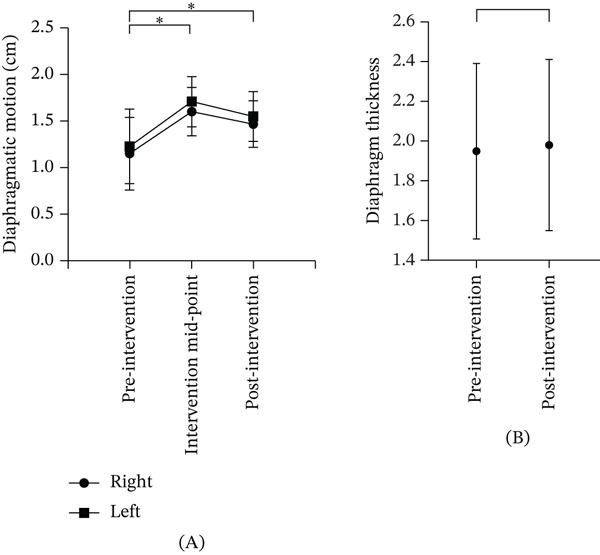
Changes in bilateral diaphragm activity and diaphragm thickness within groups.

**Figure 7:**
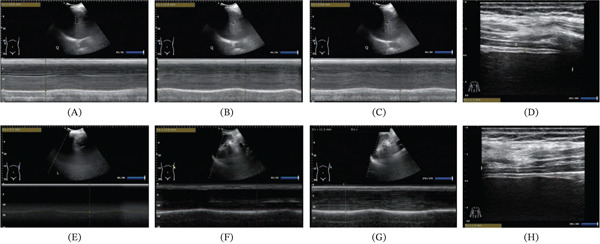
The ultrasound image shows the changes in bilateral diaphragm activity and diaphragm thickness.

EA stimulation of the phrenic nerve in the exposure group significantly increased bilateral diaphragmatic activity both during and after the intervention compared with baseline (Table 4). Statistically significant improvements were observed at midintervention (right diaphragm: *p* = 0.047; left diaphragm: *p* = 0.048) and postintervention (right diaphragm: *p* = 0.041; left diaphragm: *p* = 0.048), as shown in Figure 6A. Although diaphragmatic activity showed a slight decline after completion of EA therapy compared with midtreatment measurements, postintervention values remained higher than baseline levels, indicating a sustained functional improvement. Quantitative evaluation further demonstrated that diaphragmatic excursion increased following the intervention, with an absolute mean difference of 0.32 cm on both the right side (95% CIs: 0.18–0.46) and left side (95% CIs: 0.17–0.47). In contrast, diaphragm thickness exhibited minimal change (absolute difference: 0.03 mm; 95% CIs: −0.15 to 0.21), suggesting that EA primarily improved diaphragmatic functional mobility rather than inducing measurable structural alterations (Table 4). Consistent with these findings, diaphragmatic thickness showed no significant change from preintervention to postintervention (*p* = 0.873; Figure 6B). Detailed quantitative changes in structure and function throughout the intervention are shown in Figure 7A–H.

**Table 4:** Changes in diaphragmatic movement and thickness in exposure group.

		Preintervention	Intervention midpoint	Postintervention	Absolute difference	95% CIs
Diaphragmatic motion (cm)	Right	1.15 ± 0.39	1.60 ± 0.26	1.47 ± 0.25^∗^	0.32	[0.18, 0.46]
	Left	1.23 ± 0.40	1.71 ± 0.27	1.55 ± 0.27^∗^	0.32	[0.17, 0.47]

Diaphragm thickness (mm)		1.95 ± 0.44		1.98 ± 0.43	0.03	[−0.15, 0.21]

*Notes:* Absolute difference = postintervention − preintervention. 95% CIs calculated using paired *t*‐test method. Comparison between postintervention and preintervention in the exposure group showed differences in left diaphragmatic movement (*p* = 0.41) and right diaphragmatic movement (*p* = 0.048). Comparison of diaphragmatic thickness in the exposure group before and after the intervention showed no significant difference (*p* = 0.873).

Abbreviation: CIs, confidence intervals.

^∗^ *p* < 0.05, indicating statistical significance.

### 3.6. Adverse Events

All EA procedures were performed by experienced physicians to ensure procedural safety and consistency. During the study period, only one patient reported transient pain during EA treatment, which resolved immediately after adjustment of the needle depth without requiring further intervention. No treatment discontinuation occurred due to adverse reactions.

In addition to monitoring local discomfort related to needle insertion, patients were systematically observed throughout the intervention period for potential safety concerns, including respiratory instability, aspiration events, cardiovascular fluctuations, and other procedure‐related complications, particularly considering the vulnerable clinical status of PSP patients. No clinically significant respiratory, cardiovascular, or systemic adverse events were identified during the observation period.

## 4. Discussion

### 4.1. Limited Efficacy of Active Rehabilitation With Poor Compliance

In this retrospective cohort study, stroke was identified as a leading cause of disability and death, severely impairing patients′ daily functioning [25, 26]. Poststroke rehabilitation is a long‐term process, often complicated by high rates of comorbidities, such as pulmonary infections, pressure ulcers, urinary tract infections, and deep vein thrombosis [27–29]. In recent years, recurrent pulmonary infections have significantly hindered the rehabilitation of motor, speech, and swallowing functions in poststroke patients. As a result, researchers have sought to improve rehabilitation techniques and develop new therapeutic approaches [30].

Patients with severe motor dysfunction, especially those unable to sit or walk, experience a significant decline in daily activity, which in turn reduces cardiopulmonary function. Long‐term bedridden patients commonly present with reduced diaphragmatic movement, decreased chest wall mobility, and impaired ventilation‐perfusion function [31]. Bulbar palsy further weakens swallowing, cough reflex, and sputum clearance, leading to airway obstruction and an increased risk of pulmonary infection. In addition, advanced age, impaired consciousness, poor nutritional status, and tracheostomy use further compromise immune responses and damage the airway barrier, making the lungs more vulnerable to infection [32, 33]. Poststroke changes in the sympathetic nervous system, hypothalamic–pituitary–adrenal axis, and parasympathetic nervous system can also increase susceptibility to infections, with PNS overactivation being particularly associated with bacterial infection risk [18, 34]. The rise of multidrug–resistant and pan‐drug–resistant bacteria, coupled with the limited efficacy of antibiotics, further contributes to poor PSP outcomes. In this study, most participants were bedridden, exhibited motor and cognitive impairments, and had difficulty actively engaging in rehabilitation techniques such as airway clearance and respiratory training. As a result, these patients experienced poor pneumonia outcomes.

### 4.2. Diaphragmatic Dysfunction Severely Affects PSP Recovery

The diaphragm plays a crucial role in respiration, contributing 60%–80% of the inspiratory effort. It is innervated by the phrenic nerves, which arise from the C3 to C5 roots of the cervical plexus [15, 35, 36]. Dysfunction of the diaphragm or phrenic nerves can lead to impaired diaphragmatic function, thereby reducing respiratory efficiency. A healthy diaphragm is essential for maintaining negative intrathoracic pressure and lung compliance [19]. Hemiplegia following a stroke often leads to weakness in the trunk muscles, particularly the diaphragm, which disrupts the coordinated movement of the diaphragm that facilitates breathing. Approximately 52% of poststroke patients exhibit diaphragmatic dysfunction, which significantly compromises their respiratory function [37, 38]. Studies have shown reduced forced vital capacity, forced expiratory volume in 1 s, peak expiratory flow, and diaphragmatic movement amplitude in these patients [20]. Diaphragmatic movement is closely linked to lung ventilation and sputum clearance, both of which are critical for recovery in PSP patients. Factors such as stroke severity, including the size and location of the lesion and the patient′s NIHSS score, significantly influence the recovery of PSP [17]. In this study, participants with high NIHSS scores exhibited reduced diaphragmatic activity, further highlighting the importance of evaluating diaphragmatic function to protect against pulmonary complications, such as pneumonia and respiratory failure [39, 37].

### 4.3. EA Stimulation of the Phrenic Nerve Improves Diaphragmatic Activity

EA combines traditional acupuncture with low‐level electrical stimulation to activate both muscles and nerves. This technique activates afferent nerve fibers innervating the skin and muscles, influencing somatic and visceral functions [40]. The therapeutic effects of acupuncture are partly mediated through somatic–autonomic reflexes, which activate sympathetic and parasympathetic neural pathways [14].

From an anatomical perspective, the acupoints Tianding (LI17) and Wuyi (ST5) are located in close proximity to the cervical region, where the phrenic nerve originates (C3–C5) and courses along the anterior scalene muscle. Electrical stimulation in this region may indirectly modulate phrenic nerve excitability through adjacent neuromuscular and autonomic structures, thereby providing a plausible anatomical substrate for influencing diaphragmatic function.

Building upon this anatomical rationale, previous studies have demonstrated that EA can stimulate localized muscle training and improve the functional performance of smooth and skeletal muscles, including pelvic floor muscles [41] and renal smooth muscle [42]. In poststroke patients with tracheostomy, EA stimulation of back‐shu points has been shown to significantly enhance diaphragmatic activity and contraction velocity [19], further supporting its neuromodulatory potential in respiratory regulation.

In addition, discontinuous waveforms—characterized by strong neurostimulatory properties—have been widely adopted in clinical practice to enhance neural responsiveness [43]. The phrenic nerve, which is a mixed motor‐sensory nerve, contains approximately two‐thirds efferent fibers that control diaphragmatic movement and one‐third afferent fibers involved in sensory feedback [44, 45].

Within this neurophysiological framework, the application of EA using excitatory pulse waveforms in the present study likely facilitated both efferent motor activation and afferent sensory modulation of the phrenic nerve. This dual mechanism may contribute to enhanced diaphragmatic excursion and improved respiratory coordination. Consistent with this hypothesis, ultrasound monitoring demonstrated a significant increase in diaphragmatic movement both during and after EA intervention, indicating a sustained improvement in diaphragmatic functional activity.

### 4.4. EA Stimulation of the Phrenic Nerve Improves PSP: Possible Mechanisms

It should be emphasized that the proposed neurophysiological and neuroimmune mechanisms remain hypothetical within the context of this study, as direct measurements of autonomic activity, nerve conduction, or immune pathway modulation were not performed. Therefore, the following mechanistic interpretations should be understood as biologically plausible explanatory frameworks derived from existing literature rather than experimentally validated causal mechanisms.

This study demonstrated that EA applied at specific points along the phrenic nerve using defined excitatory frequencies led to significant improvements in diaphragmatic activity. The results showed that patients in the exposure group experienced notable improvements in pneumonia recovery compared with the nonexposure group. EA afferent stimulation of the phrenic nerve recruits motor neurons, enhancing diaphragmatic movement, which subsequently improves lung ventilation, facilitates sputum clearance, and promotes inflammation resolution.

Previous research has shown that afferent phrenic neurons can trigger physiological responses, such as increased sympathetic outflow, enhanced ventilation, and greater intercostal muscle activity [46, 47]. Additionally, phrenic afferent neurons can also activate cortical somatosensory neurons, which may play a role in respiratory perception and regulate the emotional response to pulmonary inflammation [16, 48]. In poststroke patients with impaired consciousness, cognitive decline, weakened cough reflexes, and speech disorders, stimulation of the phrenic nerve may reflexively activate cortical somatosensory neurons. After effective EA stimulation, this peripheral‐to‐central feedback loop can improve the patient′s respiratory perception and regulation. Even after treatment cessation, the reactivation of central sensory function may persist, contributing to the continued improvement in respiratory function observed in the exposure group.

In recent years, the interaction between the nervous and immune systems has gained increasing attention, particularly in studies of excessive inflammation in various diseases [49]. The nervous system communicates with immune cells through neurotransmitters and neuropeptides, whereas immune cells are influenced by neural activity. Studies of acute inflammatory lung injury and pulmonary infections have shown that neuroimmune regulation can inhibit inflammatory responses, helping to treat pneumonia and lung damage [50]. The phrenic nerve, in addition to its motor and sensory functions, contains autonomic fibers from the cervical sympathetic trunk and stellate ganglia [51]. EA applied to points near the cervical sympathetic nerve using a 2‐Hz frequency has been shown to improve peak expiratory flow and vital capacity, thereby enhancing respiratory function [52, 53]. Accordingly, EA stimulation in the present study may have influenced both motor respiratory output and autonomic regulatory pathways. Through combined mechanical and electrical activation of these neural structures, EA may contribute to modulation of neuroimmune responses and improvement of pulmonary inflammation in PSP [49].

### 4.5. Clinical Implications and Potential Role in Discharge Decision‐Making

Although this study evaluated short‐term clinical and physiological outcomes, the significant improvements observed in the intervention group hold meaningful implications for future clinical practice. In the standard management of PSP, discharge decisions are typically based on established infection control criteria, such as symptom resolution, normalized CPIS, reduction of inflammatory biomarkers, and improvement in respiratory function.

Patients receiving EA‐mediated phrenic nerve stimulation in this study exhibited greater reductions in CPIS, more rapid normalization of systemic inflammatory markers, and enhanced diaphragmatic activity as measured by ultrasound. These outcome measures directly correspond to key clinical indicators used to assess treatment response and discharge readiness in pneumonia management. Consequently, our findings suggest that diaphragmatic activity, alongside CPIS and inflammatory biomarkers, may serve as useful physiological markers to inform and potentially expedite discharge decisions in clinical settings.

Although the present findings suggest potential clinical relevance, EA‐mediated phrenic nerve stimulation should currently be interpreted as an exploratory adjunctive therapeutic approach rather than a validated tool for discharge prediction. Given the observational nature of this study and the absence of standardized discharge protocols, the results should be viewed as hypothesis‐generating rather than practice‐defining.

Importantly, clinically meaningful outcomes—including mortality, pneumonia recurrence, requirement for mechanical ventilation, and length of hospital stay—were not systematically evaluated in the present study. The absence of these endpoints limits the ability to determine whether the observed physiological improvements translate into tangible real‐world clinical benefits.

It should also be noted that discharge timing was not predefined, and uniform discharge criteria were not applied across patients. Therefore, future well‐designed prospective trials should incorporate clinically relevant outcome measures—such as length of hospital stay and time to meet predefined discharge eligibility criteria—to determine whether EA‐based phrenic nerve stimulation can safely facilitate earlier discharge without compromising patient outcomes or increasing the risk of pneumonia recurrence. In addition, future investigations exploring alternative EA targets, optimized stimulation parameters, and individualized neuromodulation strategies may further enhance respiratory recovery and overall functional outcomes in stroke populations.

### 4.6. Limitations

Several limitations should be considered when interpreting the findings of this study. First, as a retrospective analysis using real‐world clinical data, it remains susceptible to selection bias and residual confounding despite the application of PSM to adjust for baseline differences. In addition, several clinically relevant prognostic factors for PSP—such as baseline respiratory function, aspiration risk, dysphagia severity, and potential variations in antibiotic treatment strategies—were not incorporated into the propensity score model due to inherent constraints associated with retrospective data collection. The absence of these unmeasured variables may have partially influenced treatment outcomes and limits the completeness of confounding adjustment. Second, the sample size, even after matching, is relatively modest, which may constrain the statistical power and broader applicability of the results. Third, treatment allocation was not randomized but determined by clinical decision and patient preference, raising the possibility of indication bias. Fourth, outcome assessors were not blinded to group assignment, which could introduce measurement bias.

Importantly, the findings of this study should be interpreted as preliminary and hypothesis‐generating. Although the observed associations suggest potential clinical benefits of EA‐mediated phrenic nerve stimulation, they do not establish definitive causal relationships. These limitations underscore the need for cautious interpretation of the results and highlight the exploratory nature of the present study. To more conclusively establish the efficacy of EA in PSP and to clarify its role in clinical practice, future research should prioritize well‐designed prospective studies. In particular, adequately powered randomized controlled trials incorporating standardized intervention protocols, blinded outcome assessment, comprehensive clinically relevant endpoints, and longer follow‐up durations will be essential to validate these preliminary findings and refine therapeutic strategies.

## 5. Conclusion

This retrospective cohort study demonstrates that adjunctive EA, which stimulates the phrenic nerve, significantly improves diaphragmatic activity and yields superior clinical and inflammatory outcomes in patients with PSP when compared with standard care alone. These results support the therapeutic potential of EA as a beneficial passive intervention for PSP, particularly for patients with limited ability to participate in active rehabilitation. Given the exploratory nature of this study, the present findings represent an initial step toward understanding the therapeutic potential of EA in PSP. As such, they should be interpreted as hypothesis‐generating rather than conclusive. The observed improvements provide a rationale for future well‐designed randomized controlled trials to confirm therapeutic effectiveness, elucidate underlying mechanisms, and evaluate its impact on key clinical outcomes such as length of hospital stay.

Beyond improving infection control and respiratory function, future prospective studies should investigate whether these objective improvements can be systematically integrated into standardized discharge protocols and safely reduce the length of hospitalization for patients with PSP.

**Nomenclature**
PSP: poststroke pneumonia
EA: electroacupuncture
PSM: propensity score matching
NIHSS: National Institutes of Health Stroke Scale
CPIS: Clinical Pulmonary Infection Score
WBC: white blood cell count
CRP: C‐reactive protein
IL‐6: interleukin‐6
PCT: procalcitonin

## Author Contributions

Bing‐Feng Xing and Xiao‐Qiang Huang contributed equally to this study.

## Funding

No funding was received for this manuscript.

## Conflicts of Interest

The authors declare no conflicts of interest.

## Data Availability

The data that support the findings of this study are available from the corresponding author upon reasonable request.
